# First report of *Rickettsia raoultii* and *R. slovaca* in *Melophagus ovinus,* the sheep ked

**DOI:** 10.1186/s13071-016-1885-7

**Published:** 2016-11-25

**Authors:** Dan Liu, Yuan-Zhi Wang, Huan Zhang, Zhi-Qiang Liu, Ha-zi Wureli, Shi-Wei Wang, Chang-Chun Tu, Chuang-Fu Chen

**Affiliations:** 1 College of Animal Science and Technology, Shihezi University, Shihezi, Xinjiang Uygur Autonomous Region 832002 China; 2 School of Medicine, Shihezi University, Shihezi, Xinjiang Uygur Autonomous Region 832002 China; 3 Institute of Veterinary Medicine, Xinjiang Academy of Animal Science, Urumqi, Xinjiang Uygur Autonomous Region 830000 People’s Republic of China; 4 College of Animal Science, Tarim University, Alar, Xinjiang Uygur Autonomous Region 843300 China; 5 Institute of Veterinary Sciences, Academy of Military Medical Sciences, Jilin, Changchun 130062 China

**Keywords:** *Melophagus ovinus*, *Rickettsia raoultii*, *Rickettsia slovaca*, China

## Abstract

**Background:**

*Melophagus ovinus* (Diptera: Hippoboscidae), a hematophagous ectoparasite, is mainly found in Europe, Northwestern Africa, and Asia. This wingless fly infests sheep, rabbits, and red foxes, and causes inflammation, wool loss and skin damage. Furthermore, this parasite has been shown to transmit diseases, and plays a role as a vector. Herein, we investigated the presence of various *Rickettsia* species in *M. ovinus*.

**Methods:**

In this study, a total of 95 sheep keds were collected in Kuqa County and Alaer City southern region of Xinjiang Uygur Autonomous Region, northwestern China. First, collected sheep keds were identified on the species level using morphological keys and molecular methods based on a fragment of the 18S ribosomal DNA gene (18S rDNA). Thereafter, to assess the presence of rickettsial DNA in sheep keds, the DNA of individual samples was screened by PCR based on six *Rickettsia*-specific gene fragments originating from six genes: the 17-kilodalton antigen gene (*17-kDa*), 16S rRNA gene (*rrs*), surface cell antigen 4 gene (*sca4*), citrate synthase gene (*gltA*), and outer membrane protein A and B genes (*ompA* and *ompB*). The amplified products were confirmed by sequencing and BLAST analysis (https://blast.ncbi.nlm.nih.gov/Blast.cgi?PROGRAM=blastn&PAGE_TYPE=BlastSearch&LINK_LOC=blasthome).

**Results:**

According to its morphology and results of molecular analysis, the species was identified as *Melophagus ovinus*, with 100% identity to *M. ovinus* from St. Kilda, Australia (FN666411). DNA of *Rickettsia* spp. were found in 12 *M. ovinus* samples (12.63%, 12/95). *Rickettsia raoultii* and *R. slovaca* were confirmed based on phylogenetic analysis, although the genetic markers of these two rickettsial agents amplified in this study showed molecular diversity.

**Conclusions:**

This is the first report of *R. raoultii* and *R. slovaca* DNA in *M. ovinus. Rickettsia slovaca* was found for the first time around the Taklimakan Desert located in China. This finding extends the geographical range of spotted fever group rickettsiae*.*

**Electronic supplementary material:**

The online version of this article (doi:10.1186/s13071-016-1885-7) contains supplementary material, which is available to authorized users.

## Background

*Melophagus ovinus*, also referred to as the louse fly or sheep ked, is a wingless insect that belongs to the family Hippoboscidae (Diptera: Hippoboscoidea). Sheep keds are one of the most common and economically important blood-feeding ectoparasites [1]. This wingless arthropod is roughly 4 to 6 mm long and has a small head with strong piercing mouthparts. The abdominal area is wide, and the three pairs of legs are tipped with claws. As a Palaearctic species, *M. ovinus* has a wide geographical distribution [2]. For example, *M. ovinus* is native to most part of Europe, Northwestern Africa, Mongolia and North India, and this ectoparasite has been introduced into and established in Kenya, South Africa, Japan, Australia, New Zealand and most of North America [3]. In China, a small number of reports have recorded the presence of this arthropod in Qinghai, Shandong Province, and Xinjiang Uygur Autonomous Region (XUAR) [2, 4]. Although sheep are generally considered as the main host, *M. ovinus* has been observed to infest a broader range of domestic animals (goats and dogs), and wild animals (European bisons, rabbits and red foxes) as well as human beings [2, 5, 6].

*Melophagus ovinus* lives (as adult) on the hairs or fleece of their hosts, visiting the skin to feed on blood. The life-cycle of *M. ovinus* comprises the larva, pupa, nymph and adult stages [5]. The female produces a single fully developed larva every 6–8 days that firmly attaches to the wool and becomes a puparium in 6–12 h. The puparium later develops into an adult within 19–30 days [2]. As *M. ovinus* is regarded as a permanent ectoparasite, the transfer of keds from an infested to a non-infested sheep occurs by direct contact [1]. Some studies have indicated that infestation by *M. ovinus* results in pruritus, wool loss and skin damage, due to scratching, biting and rubbing, that leads to inflammation [2, 7]. Additionally, damaged areas of the skin are entry portals for bacterial infections and cutaneous myiasis, which may result in tissue necrosis, and ultimately reduces the value of the hide [5].

*Melophagus ovinus* is a biological vector of *Trypanosoma melophagium*, an apathogenic protozoan [1, 8]. Luedke et al. reported that *M. ovinus* is able mechanically to transmit bluetongue virus, which is responsible for a severe infectious disease of ruminants [9]. Additionally, *M. ovinus* may be a carrier for two organisms, e.g. *Bartonella schoenbuchensis* and *B. chomeli*, found in the USA [10], and *Anaplasma ovis* has been detected in *M. ovinus* in Hungary [11]. Similarly, Chu et al. reported that the DNA of *Borrelia garinii* and *B. valaisiana*-related group was present in *M. ovinus* [12]. Moreover, recent reports by Kumsa et al. revealed the presence of *Acinetobacter* spp. in sheep keds in Ethiopia [13].

In 2011, Hornok et al. reported the DNA of unidentified *Rickettsia* species in *M. ovinus*, based on the presence of *Rickettsia* citrate synthase gene (*gltA*) [11]. Herein, *Rickettsia* agents were detected in *M. ovinus* using six rickettsial genetic markers, the 17-kilodalton antigen gene (*17-kDa*), *gltA*, 16S rRNA gene (*rrs*), outer membrane protein A gene (*ompA*), surface cell antigen 4 gene (*sca4*), and outer membrane protein B gene (*ompB*).

## Methods

### Study areas and animals

In April 2016, sheep keds were collected from sheep in two locations near the Taklimakan Desert in the southern region of XUAR: (1) in Wuzun Town, Kuqa County (*n* = 89) (1,070.0 m above sea level; 41°72′N, 83°06′E), and (2) in Tuokayi Town, Alaer City (*n* = 6) (1,016.0 m above sea level; 40°53′N, 81°12′E).

### Sheep ked collection and morphological identification

Each sheep ked was manually removed using forceps or by hand to avoid any damage, and were then placed into 70% ethanol for subsequent identification. All sheep keds that were collected from the same infested animal were placed into pre-labeled vials and transported to the Laboratory of the School of Medicine, Shihezi University. Photographs of the sheep keds were taken with a Leica stereo microscope M165 C (LEICA M165 C, Solms, Germany). The sex and stage were determined according to standard morphological keys [13].

### DNA extraction and molecular analyses

Prior to DNA extraction, each sheep ked specimen was rinsed twice in sterile water for 15 min and then dried on sterile filter paper. Genomic DNA was individually extracted by using the TIANamp Genomic DNA Kit (TIANGEN, Beijing, China), according to the instructions provided by the manufacturer. The DNA from each specimen was eluted in 60 μl of Tris-EDTA buffer solution and stored at -20 °C under sterile conditions to preclude contamination until the sample was used for polymerase chain reaction (PCR) analysis.

To examine the phylogenetic relationships within Hippoboscidae, all DNA samples were subjected to PCR to amplify a ~985 bp fragment of the 18S ribosomal DNA gene (18S rDNA). The primers 18S-F: 5′-GTC TCA AAG ATT AAG CCA TGC ATG-3′ and 18S-R: 5′-CTT GTT AGG TTC ACC TAC GGA AAC-3′ were used in this study (the primers were designed by Primer Premier 5.0 software). The thermocycling conditions were as follows: 95 °C for 5 min, 35 cycles at 94 °C for 40 s, 57 °C for 40 s and 72 °C for 1 min and 30 s, followed by a final extension at 72 °C for 10 min. Moreover, in parallel with each amplification reaction, a negative control (distilled water) was included. Out of all the samples, 20 PCR products were randomly purified using the TIANgel Midi Purification Kit (TIANGEN, Beijing, China) and sequenced by Sangon Biotech Co., Ltd (Shanghai, China).

### Detection of rickettsial agents and sequence analysis

Identification of *Rickettsia* spp. was performed by PCR. Primers targeted six *Rickettsia*-specific gene fragments as follows: a 434 bp fragment of the *17-kDa*, 834 bp of *gltA*, 629 bp of *ompA*, 1332 bp of *rrs*, 920 bp of *sca4*, and 865 bp of *ompB*, according to previous descriptions [14–17]. Sterile water was used as a negative control and the DNA of spotted fever group rickettsiae amplified in our laboratory was used as a positive control [18–20]. The primers and cycling conditions are shown in Additional file 1. Purification and sequencing of the PCR products were conducted as described above. Phylogenetic trees were made based on the sequence distance method using the neighbor-joining (NJ) and maximum-likelihood (ML) algorithms implemented in the Molecular Evolutionary Genetics Analysis (MEGA) 6 software [21].

## Results

**Fig. 1:**
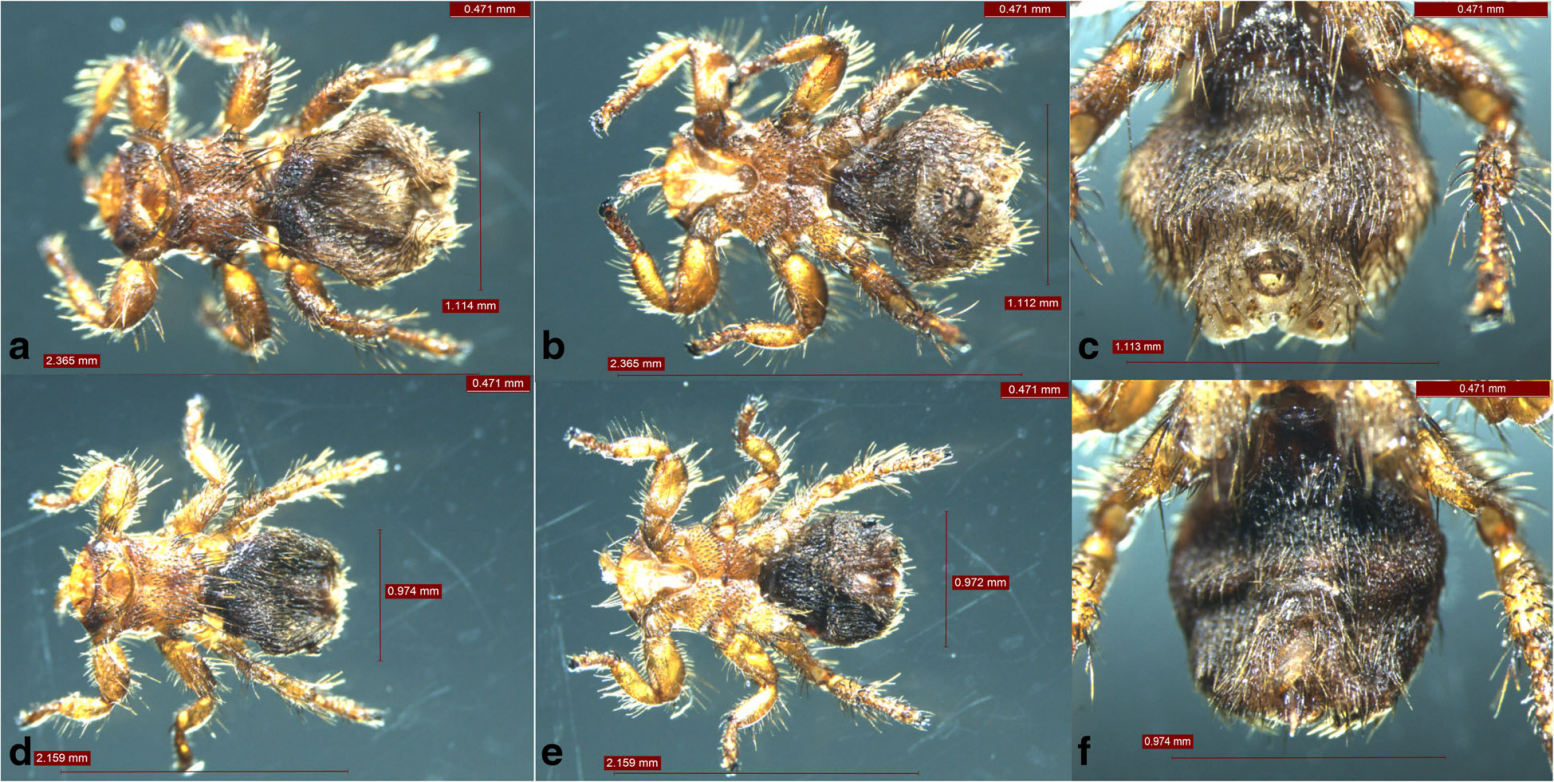
Photomicrographs of morphologically identified *Melophagus ovinus*. **a** Female, dorsal view. **b** Female, ventral view. **c** Posterior end of the female. **d** Male, dorsal view. **e** Male, ventral view. **f** Posterior end of the male

A total of 95 sheep keds were collected from three sheep flocks (*n* = 230). Morphologically, these ectoparasites were identified as *Melophagus ovinus* (Fig. 1a-f). The length of the 18S rDNA sequences amplified from *M. ovinus* was 985 bp, which was longer than the available sequences of *M. ovinus* in GenBank (FN666411, from St. Kilda, Australia). Interestingly, molecular analysis of 20 *M. ovinus* samples showed that two different lineages exist due to their diversity (99.8–100%) in 18S rDNA flanking fragments. The full length sequences from our study were deposited in GenBank (KX506727 and KX506728).

**Fig. 2:**
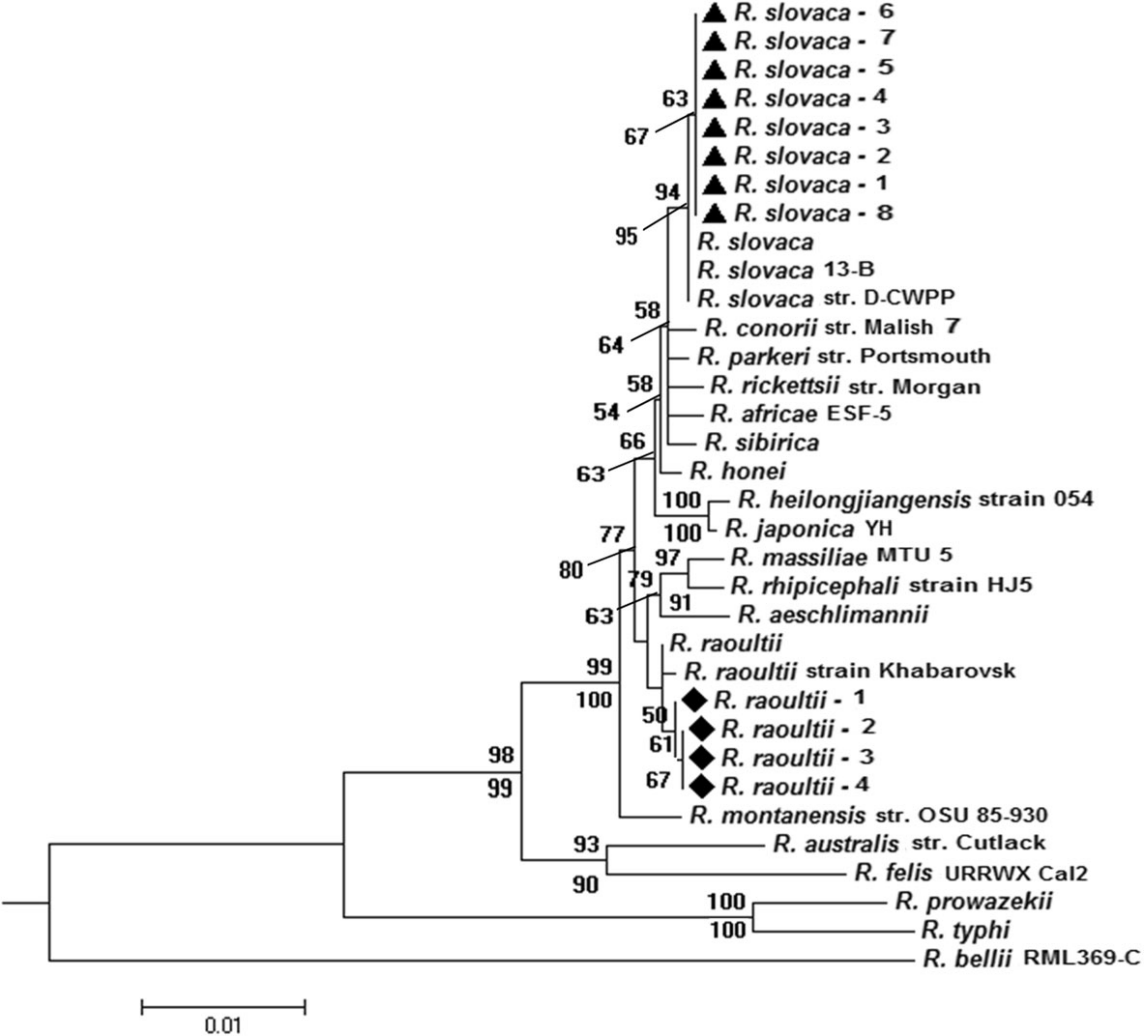
Phylogenetic tree of the *17-kDa - gltA - ompA - rrs - sca4 - ompB* concatenated sequence of *Rickettsia slovaca* (▲) and *R. raoultii* (◆) from *Melophagus ovinus* obtained in this study and sequences from *Rickettsia* species retrieved from the GenBank database. The tree was constructed on the basis of neighbor-joining (NJ; 500 bootstrap replicates) and maximum-likelihood (ML, 1,000 bootstrap replicates) analyses using MEGA6. The scale bar represents the inferred substitutions per nucleotide site. The relative support for clades in the tree was produced from the NJ and ML analyses

Out of 95 *M. ovinus* samples 12 were found to be positive for the six *Rickettsia* genetic markers (*17-kDa*, *gltA*, *ompA*, *rrs*, *sca4* and *ompB*). Out of the 12 positive samples, four were confirmed as *R. raoultii*, and the remaining eight were identified as *R. slovaca* on the basis of the *17-kDa - gltA* - *ompA - rrs - sca4 - ompB* concatenated sequence (Fig. 2)*.*

Concerning *R. raoultii*, the loci *rrs* and *17-kDa* were identical in each sample and showed identities of 100% (1,196/1,196 bp) and 99.51% (407/409 bp), respectively, when compared with the *R. raoultii* strain Khabarovsk (CP010969). However, in the remaining four genetic markers the differences appeared to be more pronounced. For example, genetic analysis revealed two different sequences for *gltA*, three sequences for *ompA*, three sequences for *ompB*, and two sequences for *sca4*.

Concerning *R. slovaca*, sequences of the *17-kDa*, *gltA*, *rrs*, and *sca4* genes appeared to be conserved, and showed similarities of 100% (411/411 bp) for *17-kDa*, 100% (834/834 bp) for *gltA*, 100% (1,196/1,196 bp) for *rrs*, and 99.88% (867/868 bp) for s*ca4* when compared with those of *R. slovaca* strain D-CWPP (CP003375). However, for both *ompA* and *ompB*, three different sequences were obtained, respectively. The detailed similarities and divergences of the sequences in this study are shown in the Additional file 2. All sequences from this study were deposited in the GenBank database (*16S*: KX506722–KX506723; *17-KDa*: KX506725–KX506726; *gltA*: KX506730–KX506732; *ompA*: KX506733–KX506738; *ompB*: KX506739–KX506744; *sca4*: KX506745–KX506747).

## Discussion

An overall prevalence of *Rickettsia* spp. in *M. ovinus* collected from sheep in southern XUAR was 12.63% (12/95). In these samples the presence of *R. raoultii* and *R. slovaca* DNA was confirmed by conventional PCR followed by sequencing. To the best of our knowledge, this is the first molecular evidence of *R. raoultii* and *R. slovaca* DNA in *M. ovinus*.

*Rickettsia raoultii* was first identified in 1999 [22]. In the past five years, this species has been detected (among the others) in Mongolia, Georgia, Germany, Slovakia and China [23–27]. To date, the DNA of *R. raoultii* was shown to be present in 14 tick species of the genera *Dermacentor*, *Rhipicephalus*, *Haemaphysalis*, *Hyalomma*, *Ixodes* and *Amblyomma* [18]. *Rickettsia slovaca*, a member of the spotted fever group (SFG) rickettsiae, was first isolated in 1968 from *Dermacentor marginatus* in Slovakia [28], and later on described as the causative agent of tick-borne lymphadenopathy [29]. This species has been detected subsequently in Georgia [24], Germany [25], Greece and Turkey [30, 31]. In 2012, *R. slovaca* and *R. raoultii* were reported for the first time in *Dermacentor silvarum* ticks in XUAR, northwest China [32]. However, *R. raoultii* has seldom been reported in other arthropods. In this study, molecular evidence is provided for the presence of *R. raoultii* DNA in sheep keds (*M. ovinus*).

In a previous study, an unidentified *Rickettsia* species was detected in *M. ovinus* collected in Hungary, with a prevalence of 1.67% (1/60) based on the *gltA* gene detection [11]. Recently, two reports have shown the absence of *Rickettsia* spp. in *M. ovinus* collected in Ethiopia and the Czech Republic [13, 33].

Herein, a high prevalence (12.63%, 12/95) of *R. raoultii* and *R. slovaca* DNA was demonstrated in *M. ovinus* from the Taklimakan Desert in China. The observations from this study have extended the spectrum of pathogens potentially present in *M. ovinus*. Future investigations are warranted to elucidate the genetic diversity of *R. raoultii* and *R. slovaca* in *M. ovinus*. Additionally, the presence of these *Rickettsia* spp. should be examined in a broader range of arthropods.

## Conclusions

This is the first report of genetic markers of *R. raoultii* and *R. slovaca* in *M. ovinus. Rickettsia slovaca* was found for the first time around the Taklimakan Desert located in China. This findings extend our knowledge on the geographical distribution of spotted fever group rickettsiae*.*

## Additional files

Additional file 1: PCR protocol for the detection of *Rickettsia* spp., Xinjiang, China. (DOCX 22 kb)
Additional file 2: Closest relative sequences of the *Rickettsia raoultii* and *Rickettsia slovaca* detected in the sheep keds (*Melophagus ovinus*), Northwest of China. (DOC 109 kb)

## Abbreviations

*17-kDa*: 17-kilodalton antigen gene
*gltA*: Citrate synthase gene
MEGA: Molecular evolutionary genetics analysis
ML: Maximum-likelihood
NJ: Neighbor-joining
*ompA*: Outer membrane protein A gene
*ompB*: Outer membrane protein B gene
PCR: Polymerase chain reaction
*rrs*: 16S rRNA gene
*sca4*: Surface cell antigen 4 gene
SFG: Spotted fever group
XUAR: Xinjiang Uygur Autonomous Region
