# Supplementary material for: First report of Rickettsia raoultii and R. slovaca in Melophagus ovinus, the sheep ked
Source: Parasit Vectors. 2016 Nov 25;9:600. doi: 10.1186/s13071-016-1885-7 (PMC5123371; doi:10.1186/s13071-016-1885-7)
Supplement: Additional file 1: — PCR protocol for the detection of Rickettsia spp., Xinjiang, China. (DOCX 22 kb) [file 13071_2016_1885_MOESM1_ESM.docx]

**Additional file 1**

**PCR protocol for the detection of *Rickettsia* spp., Xinjiang, China**

The PCR equipment was a TechneTC-412 thermal cycler, Barloworld Scientific, Cambridge, UK.

**1. PCR amplification to detect Rickettsia spp. based on the *17-kDa* gene**

Each reaction consisted of 1 μL of sheep keds genomic DNA (50 ng) and 12.5 μL of a PCR mix containing 50 mM KCl, 10 mM Tris-HCl (pH 8.3), 1.5 mM MgCl_2_, 250 μM of each dNTP, 40 pmol of each primer (*17-kDa*-5*,* *17-kDa*-3), and 1.0 U of *Taq* DNA polymerase (TaKaRa Taq Version 2.0, Takara, Dalian, China).The cycling conditions consisted of an initial 5-min denaturation at 95°C, followed by 33 cycles at 95°C for 30 s, 61°C for 30 s, and 72°C for 30 s, with a final extension at 72°C for 8 min. After the first amplification, 1µL (10x diluted) of the product was reamplified, now using inner primers *17-kDa*-1 and *17-kDa*-2. The amplification program was the same with above.

**2. PCR amplification to detect *Rickettsia spp.* based on the *gltA* gene**

The PCR amplifications were performed in a 25-μL reaction volume. The reaction mixture contained 0.75 μmol/L of each primer (*gltA*), 250 μM of each dNTP, and 1.0 U of *Taq* DNA polymerase (TaKaRa Taq Version 2.0, Takara, Dalian, China). The cycling conditions consisted of an initial 5-min denaturation at 95°C, followed by 40 cycles at 95°C for 30 s, 52°C for 30 s, and 72°C for 40 s, with a final extension at 72°C for 5 min.

**3. PCR amplification to detect *Rickettsia spp.* based on the *ompA* gene**

The PCR amplifications were performed in a 25-μL reaction volume. The reaction mixture contained 0.75 μmol/L of each primer (*ompA*), 250 μM of each dNTP, and 1.0 U of *Taq* DNA polymerase (TaKaRa Taq Version 2.0, Takara, Dalian, China). The cycling conditions consisted of an initial 5-min denaturation at 95°C, followed by 35 cycles at 95°C for 30 s, 55°C for 45 s, and 72°C for 45 s, with a final extension at 72°C for 8 min.

**4. PCR amplification to detect *Rickettsia spp.* based on the *ompB* gene**

The PCR amplifications were performed in a 25-μL reaction volume. The reaction mixture contained 0.75 μmol/L of each primer (*ompB*), 250 μM of each dNTP, and 1.0 U of *Taq* DNA polymerase (TaKaRa Taq Version 2.0, Takara, Dalian, China). The cycling conditions consisted of an initial 5-min denaturation at 95°C, followed by 40 cycles at 95°C for 30 s, 52°C for 30 s, and 72°C for 1 min, with a final extension at 72°C for 8 min.

**5. PCR amplification to detect *Rickettsia spp.* based on the *rrs* gene**

The PCR amplifications were performed in a 25-μL reaction volume. The reaction mixture contained 0.75 μmol/L of each primer (*rrs*), 250 μM of each dNTP, and 1.0 U of *Taq* DNA polymerase (TaKaRa Taq Version 2.0, Takara, Dalian, China). The cycling conditions consisted of an initial 5-min denaturation at 95°C, followed by 37 cycles at 95°C for 45 s, 58°C for 40 s, and 72°C for 1 min 20 s, with a final extension at 72°C for 5 min.

**6. PCR amplification to detect *Rickettsia spp.* based on the *sca4* gene**

The PCR amplifications were performed in a 25-μL reaction volume. The reaction mixture contained 0.75 μmol/L of each primer (*sca4*), 250 μM of each dNTP, and 1.0 U of *Taq* DNA polymerase (TaKaRa Taq Version 2.0, Takara, Dalian, China). The cycling conditions consisted of an initial 5-min denaturation at 95°C, followed by 37 cycles at 95°C for 30 s, 55°C for 30 s, and 72°C for 1 min, with a final extension at 72°C for 8 min.

Nucleotide sequences of the primers used for the identification *Rickettsia spp.*

| Gene | Primer | Sequence(5’-3’) | Reference |
| --- | --- | --- | --- |
| *17-kDa* | 17K-5 | GCTTTACAAAATTCTAAAAACCATATA | [1] |
|  | 17K-3 | TGTCTATCAATTCACAACTTGCC | [1] |
|  | 17KD1 | GCTCTTGCAACTTCTAT GTT | [1] |
|  | 17KD2 | CATTGTTCGTCAGGTTGGCG | [1] |
| *rrs* | Rick-16S-F3 | ATCAGTACGGAATAACTTTTA | [1] |
|  | Rick-16S-R4 | TGCCTCTTGCGTTAGCTCAC | [1] |
| *gltA* | CS-239 | GCTCTTCTCATCCTATGGCTATTAT | [2] |
|  | CS-1069 | GAGGGTCTTCGTGCATTTCTT | [2] |
| *ompB* | 120-M59 | CCGCAGGGTTGGTAACTGC | [2] |
|  | 120-807 | CCTTTTAGATTACCGCCTAA | [2] |
| *ompA* | 190-70F | ATGGCGAATATTTCTCCAAAA | [3] |
|  | 190-701R | GTTCCGTTAATGGCAGCATCT | [3] |
| *sca4* | ScaD-F | CGGTAACCTAGATACAAGTGA | [4] |
|  | ScaD-R | TATAAGCTATTGCGTCATCTC | [4] |

References

1. Anstead CA, Chilton NB. A novel Rickettsia species detected in Vole Ticks (Ixodes angustus) from Western Canada. Appl Environ Microbiol. 2013; 79(24): 7583-9.
2. Mcintosh D, Bezerra RA, Luz HR, Faccini JLH, Gaiotto FA, Gine GAF, et al. Detection of Rickettsia bellii and Rickettsia amblyommii in Amblyomma longirostre (Acari: Ixodidae) from Bahia state, Northeast Brazil. Brazilian Journal of Microbiology. 2015; 46(3): 879-83.
3. Zhang L, Jin J, Fu X, Raoult D, Fournier PE. Genetic Differentiation of Chinese Isolates of Rickettsia sibirica by Partial ompA Gene Sequencing and Multispacer Typing. J Clin Microbiol. 2006; 44(7): 2465-7.
4. Zhao SS, Li HY, Yin XP, Liu ZQ, Chen CF, Wang YZ . First detection of Candidatus Rickettsia barbariae in the flea Vermipsylla alakurt from north-western China. Parasit Vectors. 2016; 9(1): 1-5.
