# Supplementary material for: First report of Rickettsia raoultii and R. slovaca in Melophagus ovinus, the sheep ked
Source: Parasit Vectors. 2016 Nov 25;9:600. doi: 10.1186/s13071-016-1885-7 (PMC5123371; doi:10.1186/s13071-016-1885-7)
Supplement: Additional file 2: — Closest relative sequences of the Rickettsia raoultii and Rickettsia slovaca detected in the sheep keds (Melophagus ovinus), Northwest of China. (DOC 109 kb) [file 13071_2016_1885_MOESM2_ESM.doc]

**Additional file 2**

Closest relative sequences to the partial *17-kDa*, *16S*, *gltA*, *ompA*, *ompB* and *sca4* genes, sequences of the *Rickettsia raoultii*(Additional file 2A), *Rickettsia slovaca* (Additional file 2B) detected in the sheep keds (*Melophagus ovinus* ), Northwest of China.

| Gene | *Rickettsia* (GenBank accession No.) | % Sequence similarity(bp) |
| --- | --- | --- |
| **Additional file 2A** | | |
| *17-kDa* ( KX506726) | *Rickettsia raoultii* strain Khabarovsk (CP010969)  *Rickettsia raoultii* isolate MDJ3 (JX885457)  *Rickettsia raoultii* strain Alashankou-131 (KT261760) | 99.51 (407/409)  99.51 (407/409)  99.49 (394/396) |
| *16S (KX506723*) | *Rickettsia raoultii* isolate BL029-2  ( KJ410261)  *Rickettsia raoulti*i isolate TC250-11 (KJ410259)  *Rickettsia raoultii* isolate TC249-10 (KJ410258) | 100 (1196/1196)  100 (1196/1196)  100 (1196/1196) |
| *gltA* (KX506731)  *gltA* (KX506732) | *Rickettsia raoultii* strain Khabarovsk (CP010969)  *Rickettsia raoultii* (KU310590) | 100 (823/823)  100 (823/823) |
|  | *Rickettsia raoulti*i (KU310589)  *Rickettsia raoultii* strain Khabarovsk (CP010969)  *Rickettsia raoultii* (KU310590)  *Rickettsia raoultii* (KU310589) | 100 (823/823)  99.88 (833/834)  99.88 (833/834)  99.88 (833/834) |
| *ompA* (KX506736)  *ompA* (KX506737)  ompA (KX506738) | *Rickettsia raoultii* strain WB16/Dm Monterenzio (HM161789) | 94.44 (594/629) |
|  | *Rickettsia raoultii* strain WB14/Dm R Casola Valsenio (HM161792)  *Rickettsia raoultii* strain Khabarovsk (CP010969)  *Rickettsia raoultii* strain WB16/Dm Monterenzio (HM161789)  *Rickettsia raoultii* strain WB14/Dm R Casola Valsenio (HM161792)  *Rickettsia raoultii* strain Khabarovsk (CP010969) | 94.28 (593/629)  93.96 (591/629)  100 (629/629)  99.84 (628/629)  99.52 (626/629) |
|  | *Rickettsia raoultii* strain Khabarovsk (CP010969)  *Rickettsia raoultii* strain WB16/Dm Monterenzio (HM161789)  *Rickettsia raoultii* strain WB14/Dm R Casola Valsenio (HM161792) | 99.84 (628/629)  99.68 (627/629)  99.52 (626/629) |
| *ompB* (KX506742)  *ompB* (KX506743)  *ompB* (KX506744) | *Rickettsia raoultii* strain Khabarovsk ( CP010969)  *Rickettsia raoultii* strain Khabarovsk (DQ365798) | 99.88 (836/837)  99.88 (836/837) |
|  | *Rickettsia raoultii* (KU310593)  *Rickettsia raoultii* (KU310593)  *Rickettsia raoultii* (KU310592)  *Rickettsia raoultii* strain Marne (DQ365797)  *Rickettsia raoultii* (KU310593)  *Rickettsia raoultii* (KU310592)  *Rickettsia raoultii* strain Marne (DQ365797) | 99.76 (835/837)  99.88 (836/837)  99.88 (836/837)  99.88 (836/837)  100 (837/837)  100 (837/837)  100 (837/837) |
| *Sca4* (KX506746)  *Sca4* (KX506747) | *Rickettsia raoultii* strain DRET2 (JN242188)  *Rickettsia raoultii* strain Khabarovsk (CP010969)  *Rickettsia raoultii* strain Marne (DQ365807)  *Rickettsia raoultii* strain Khabarovsk (CP010969)  *Rickettsia raoultii* strain Elanda-23/95 (EU036983)  *Rickettsia raoultii* strain Khabarovsk (DQ365808) | 99.31 (864/870)  99.08 (871/879)  99.20 (864/871)  99.77 (877/879)  99.77 (868/870)  99.77 (868/870) |
| **Additional file 2B** | | |
| *17-kDa* (KX506725) | *Rickettsia slovaca* str. D-CWPP (CP003375)  *Rickettsia slovaca* 13-B (CP002428)  *Rickettsia slovaca* clone 50 (JN182788) | 100 (411/411)  100 (411/411)  100 (389/389) |
| *16S* (KX506722) | *Rickettsia slovaca* str. D-CWPP (CP003375)  *Rickettsia slovaca* isolate TC250-17 (KJ410262)  *Rickettsia slovaca* 13-B strain 13-B (NR074462) | 100 (1196/1196)  100 (1196/1196)  100 (1196/1196) |
| *gltA* (KX506730) | *Rickettsia slovaca* str. D-CWPP (CP003375)  *Rickettsia slovaca* (CP002428)  *Rickettsia slovaca* N.A. 13-B (RSU59725) | 100 (834/834)  100 (834/834)  100 (834/834) |
| *ompA* ( KX506733) | *Rickettsia slovaca* str. D-CWPP (CP003375 ) | 100 (632/632) |
|  | *Rickettsia slovaca* 13-B (CP002428)  *Rickettsia slovaca* strain WB18/Dm Monterenzio (HM161786) | 100 (632/632)  100 (632/632) |
| *ompA* (KX506734)  *ompA* (KX506735) | *Rickettsia slovaca* str. D-CWPP (CP003375)  *Rickettsia slovaca* 13-B (CP002428)  *Rickettsia slovaca* strain WB18/Dm Monterenzio (HM161786)  *Rickettsia slovaca* str. D-CWPP (CP003375)  *Rickettsia slovaca* 13-B (CP002428)  *Rickettsia slovaca* strain WB18/Dm Monterenzio (HM161786) | 98.89 (625/632)  98.89 (625/632)  98.89 (625/632)  99.84 (631/632)  99.84 (631/632)  99.84 (631/632) |
| *ompB* (KX506739)  *ompB* (KX506740)  *ompB* (KX506741) | *Rickettsia slovaca* str. D-CWPP (CP003375)  *Rickettsia slovaca* 13-B (CP002428)  *Rickettsia slovaca* (AF123723) | 99.76 (835/837)  99.76 (835/837)  99.76 (835/837) |
|  | *Rickettsia slovaca str. D-CWPP* (CP003375)  *Rickettsia slovaca* 13-B (CP002428)  *Rickettsia slovaca* (AF123723) | 99.88(836/837)  99.88(836/837)  99.88(836/837) |
|  | *Rickettsia slovaca str. D-CWPP* (CP003375)  *Rickettsia slovaca* 13-B (CP002428)  *Rickettsia slovaca* (AF123723) | 100 (837/837)  100 (837/837)  100 (837/837) |
| *Sca4* (KX506745) | *Rickettsia slovaca* str. D-CWPP (CP003375)  *Rickettsia slovaca* 13-B(CP002428)  *Rickettsia slovaca* (AF155054) | 99.88 (867/868)  99.88 (867/868)  99.88(865/866) |
